# Driving Skills Tested on Simulator After Strabismus Surgery: A Prospective Study

**DOI:** 10.1167/tvst.9.8.36

**Published:** 2020-07-24

**Authors:** Dan Derhy, Ségolène Lithfous, Claude Speeg, David Gaucher, Olivier Despres, André Dufour, Tristan Bourcier, Arnaud Sauer

**Affiliations:** 1 Department of Ophtalmologie, Centre Hospitalier Universitaire (CHU) – Université de Strasbourg, Nouvel Hôpital Civil, Strasbourg, France; 2 CI2N, Centre d'Investigations Neurocognitives & Neurophysiologiques UMS 3489 CNRS / UdS . Strasbourg, France

**Keywords:** strabismus surgery, driving simulator, visual field

## Abstract

**Purpose:**

The sense of vision is responsible for 90% of the information obtained by the motorist. Improvement in binocular visual acuity (VA) and visual field (VF) achieved after strabismus surgery could have beneficial effects on driving. Our study sought to identify functional improvements (VA and VF) and improvements in driving ability following strabismus surgery.

**Methods:**

In a prospective cohort study, the following parameters are analyzed before and 3 months after strabismus surgery: simulated driving performance (including eye movements and actions on vehicle control), binocular VA, binocular VF, and self-confidence during driving.

**Results:**

Twenty patients participated in the study. The mean preoperative logMAR binocular VA and stereopsis do not significantly differ from the postoperative. The mean Esterman VF score increases from 91.3 (±17.2) preoperatively to 96.9 (±13.9) postoperatively (*P* = 0.045). The mean self-confidence directed at driving scores decreases from 20.5 (±10.3) points before surgery to 11.0 (±6.0) points after surgery (*P* < 0.001). The distance at which the road signs are identified is significantly higher after surgery. The average speed of the vehicle and the speed near the targets (30 m) increase significantly after strabismus surgery. A significant decrease in ocular movements near targets is also observed. The number of brake pedal depressions and the rate of brake pedal depressions slightly decrease after surgery.

**Conclusions:**

This study demonstrates the potential beneficial effects of strabismus surgery on driving ability, with significant improvements in self-confidence during driving, VF, and driving on a simulator.

**Translational Relevance:**

This was the first study to use a driving simulator in strabismus.

## Introduction

The sense of vision is responsible for 90% of the information obtained by the motorist.^1^ Various situations in road traffic have to be evaluated at different speeds with respect to size, distance, and position.^2^^,^^3^ Thus, visual driving ability is legally established based objective criterion such as visual acuity (VA), visual field (VF), and possibly night vision and glare criteria.^4^ Throughout life, the ability to drive may be impaired by eye diseases, such as age-related macular disease (AMD), cataract, VF defects, and glaucoma.^5^^–^^7^ Several studies objectified the driving disturbances associated with these pathologies in real life settings and also using driving simulators. Patients with cataracts have been found to be more frequently responsible for road accidents than the general population, while reducing their driving time.^8^^,^^9^ Regarding glaucoma, poor on-road driving performance with a trend to spontaneously give up driving has been observed, and simulator-based studies have revealed an increased number of eye movements and driving difficulties in particular conditions (night, fog, rain, and highway).^10^^–^^13^

Unilateral vision impairment and amblyopia can result in reduced stereopsis. Reduced stereopsis has been shown to adversely affect visuomotor tasks, such as prehension, walking, reading, and also driving.^1^^,^^6^^,^^14^ In a recent study, Baker et al. demonstrated that young adults were less likely to acquire a driver's license than those without these condition. This could be the result of a lack of confidence directed at driving. However, among licensed drivers, the risk of a police-reported crash was not increased.^2^ Patients with strabismus have a VF and stereo acuity defect. To our knowledge, no simulator-based study has investigated driving ability of patients with strabismus and following strabismus surgery. The use of a driving simulator could allow the study of different parameters, such as speed of the vehicle, position in the lane, and use of the brakes in a perfectly safe atmosphere.

As strabismus surgery is expected to increase self-confidence, stereopsis, and VF in the case of convergent strabismus.^15^^–^^17^ As previously detailed, these functional elements are involved during driving. The present pilot study aimed to explore the beneficial effects of strabismus surgery on driving using subjective criteria (self-confidence questionnaire directed at driving), objective criteria (VA and VF), and standardized driving simulator assessment before and after surgery.

## Methods

### Inclusion and Exclusion Criteria

In this prospective cohort study (Clinical trials NCT 02570555), the inclusion criteria were as follows: male or female subjects, over 18 years of age, presenting with nonparalytic strabismus (permanent deviation of > 15 diopters) that had been stable for > 1 year, seeking a surgical opinion, holding a driving license, and having signed an informed consent. Any medical condition incompatible with driving under the terms of the French Decree of December 18, 2015, relating to light vehicle driving license (category B) and dropping out of one session on the driving simulator were considered an exclusion criterion. Inclusion in the study was systematically offered to patients meeting the inclusion criteria. A total of 21 consecutive patients were included, 1 patient left the study after the first simulator session (motion sickness), no patient refused to participate in the study.

Each participant completed two sessions during the study. For patients, sessions 1 and 2 took place 1 month before and approximately 3 months after strabismus surgery, respectively.

All patients signed an explicit informed consent form. We conducted our study in compliance with recognized international standards, including the International Conference on Harmonization (ICH), the Council for International Organizations of Medical Sciences (CIOMS) and the principles of the Declaration of Helsinki. The study has been validated by an independent ethics committee.

### Outcomes

In each session, the following parameters were studied: (1) binocular VA and binocular VF, (2) self-confidence during driving measured using a self-administered self-confidence questionnaire (Driving Habits Questionnaire [DHQ] and Amblyopia and Strabismus Questionnaire [ASQE]), and (3) simulated driving performance.

#### Visual Acuity and Visual Field

In order to evaluate the potential functional improvements brought by surgery, monocular and binocular VA, stereoscopic vision (randot test), and the Esterman score using Humphrey automated perimetry were measured 1 month before and 3 months after strabismus surgery.

#### Self-Confidence Questionnaire (DHQ and ASQE)

Driving confidence was assessed using a questionnaire that was based on quality of life questionnaires designed for patients with visual disturbances and strabismus (DHQ and ASQE), including only the questions relating to driving.^18^ The questions were focused on eight items: parking, driving at night, driving in fog, driving in rain, driving on an unknown road, ability to judge distances, ability to detect obstacles, and giving up driving. The response to each item is scored from 0 (no complaint) to 5 (major difficulty leading to not delaying the task). Thus, lower scores indicate higher self-confidence during driving.

#### Driving Ability on Simulator

Driving ability was tested on the CI2N driving simulator (Center for Neurocognitive and Neurophysiological Investigations, UMS 3489 - CNRS, University of Strasbourg, France; see Supplementary Video).

The driving simulator consisted of the front portion of a car passenger cabin mounted on a mobile platform with 4 + 2 levels of freedom (longitudinal, vertical, rolling and pitching the passenger cabin, and rolling and pitching of the driver's seat) associated to a real-time interactive display unit. Eye movements, actions on vehicle controls, as well as vehicle position in the virtual environment, distance covered, and driving time and speed were recorded at 30 Hz. A three-dimensional virtual city was created using the Cityengine (Esri) software. The scenery was projected using eight Epson EB-W10 projectors at a rate of 60 frames per second and a resolution of 1280 × 800 pixels. Projections were carried out using four computers with four 2.8 GHz Intel Core i7 processors equipped with ATI Firepro V7800 graphic cards. Height projection screens (8200 × 180 cm) were placed at a distance of 241 cm around the simulator, covering a 360° horizontal and 28° vertical field of view. The virtual environment represented a typical large city with different building types forming visually distinct districts. The city was comprised of 1217 objects, including buildings, shops, monuments, street furniture, and green spaces; there were 274 intersections and 576 street segments, with a total of 4 km² covered (see Supplementary Video for a description of the driving simulator).

Participants were instructed to follow a 5 km itinerary in the virtual city, marked with signposts at intersections. From driving simulator data, both the participants’ performances in identifying the road signs and their driving performance were evaluated.

- To test identification performance during driving, road signs displaying the letters “O” or “D” were placed along the way. The participants had to detect all the “D” road signs and to press a button on the left side of the steering wheel each time they detected one while driving. On the other hand, the subjects were instructed to ignore the “O” road signs. In total, each letter was presented 11 times. During the simulator driving sessions, participants’ eye movements were recorded using an “eye-tracker” device (Smart-Eye). The identification performance of road signs of each participant was evaluated (i) by subtracting the number of false alarms (i.e. “O” road sign) from the number of correct detections (i.e. “D” road sign). This index corresponded to the subject's perceptual sensitivity while neutralizing a possible bias or response strategy. (ii) We also calculated for each participant the average distance to the target (“D” or “O” road signs) at the time of identification.
- To evaluate the driving performance, (iii) we calculated for each participant the vehicle's average speed over the entire itinerary, as well as 30, 50, and 100 meters before the targets. (iv) Eye movements were analyzed with a focus on the variation (in degrees of visual angle) of these movements throughout the driving simulator session, as well as 30, 50, and 100 meters before the targets. (v) We also calculated for each participant the rate of brake pedal depressions that provide measures of braking intensity, and the mean number of brake pedal pressures during the simulator driving task.

To distinguish between the changes in driving parameters that would be due to the result of surgical management and those due to a learning effect, we also enrolled non-strabismic controls who performed the same simulator driving test as patients with strabismus. The controls were matched in terms of age and driving experience (number of kilometer / year and driving license age).

### Statistical Analysis

For clinical data, results were presented as mean and standard deviations. Comparisons of quantitative variables were carried out using either the Student's *t*-test (for Gaussian variables), with possible corrections for heterogeneity of variances, or the non-parametric Mann—Whitney—Wilcoxon test (for non-Gaussian variables). Comparisons between more than two variables were performed by either analysis of variance (ANOVA, Gaussian variables) or its non-parametric equivalent (i.e. the Kruskal—Wallis test (non-Gaussian variables).

## Results

### Study Population

In total, 21 patients participated in this study. One patient dropped out after the first session because of motion sickness. Thus, 20 patients were included in the study. The group consisted of 10 women and 10 men. The mean age of the study population was 42.5 (±15.7) years. Regarding the participants’ experience as drivers, patients had on average 13,736 (±12,712) km per year on average over 21.7 (±14.7) years. Seven patients had convergent strabismus and 13 had divergent strabismus (6 consecutive strabismus and 7 neglected childhood strabismus).

The mean maximum angle of deviation was 40.0 prism diopters (±13.5) preoperatively and was significantly reduced postoperatively, measured at 5.0 prism diopters (±6.1). All patients had a postoperative maximum angle of deviation below 10 prism diopters. Detailed results are shown in the Table.

**Table.** Comparison of Pre- and Postoperative Visual Acuity, Visual Field, and Driving Confidence Values

Outcomes		Before Surgery	After Surgery	*P* Values
***Visual acuity and visual field measurements***				
**Visual Acuity (LogMAR)**		0.01 [0.1]	0.00 [0.1]	0.754
**Stereopsis (Sec)**		NA	400 [100] (*N* = 5)	0.371
**Esterman** **Score**	**All patients (*N* = 20)**	91.3 (±17.2)	96.9 (±13.9)	0.0450
	***Esotropia* (*N* = 7)**	*74.5 (±5.3)*	*87.6 (±10.1)*	*0.0071*
	**Exotropia (*N* = 13)**	100.6 (±13.8)	101.8 (±13.3)	0.781
***Driving confidence questionnaire***				
**Self-confidence during driving scores**		*20.5* *(±10.3)*	*11.0 (±6.0)*	*0.0012*
***Target identification performance in the driving simulator***				
**Mean differences between the number of correct identification and the number of false alarms**		8.2 (±1.5)	8.4 (±1.4)	0.665
**Mean target identification distance** **,** **meters**		*81.0 (±7.2)*	*94.7 (±5.2)*	*< 0.001*
***Driving performance in the driving simulator***				
**Mean vehicle speed** **,** **km/h**	Entire course	*36.6 (±2.8)*	*38.9 (±2.8)*	*0.0136*
	100 m of target	37.5 (±3.6)	38.2 (±3.6)	0.542
	50 m of target	37.1 (±2.8)	38.5 (±2.8)	0.122
	30 m of target	*34.8 (±4.1)*	*37.8 (±3.1)*	*0.0123*
**Eye movements** **,** **degree of visual angle**	Entire course	0.03772 (±0.005)	0.03600 (±0.005)	0.283
	100 m of target	0.03650 (±0.007)	0.03700 (±0.009)	0.846
	50 m of target	0.03650 (±0.008)	0.03550 (±0.005)	0.638
	30 m of target	*0.03700 (±0.007)*	*0.02950 (±0.009)*	*0.0072*
**Brake pedal depression**	Rate of brake pedal depression (%)	*26.8 (±1.6)*	*25.1 (±1.5)*	*0.0013*
	Brake pedal pressures (Mean number)	*39.5 (±4.2)*	*34.7 (±3.8)*	*0.0005*

### Visual Acuity and Visual Field Measurements

The mean preoperative logMAR binocular VA and stereopsis did not significantly differ from the postoperative.

The mean Esterman VF score increased from 91.3 (±17.2) preoperatively to 96.9 (±13.9) postoperatively (*P* = 0.045). For convergent strabismus, the mean Esterman VF score increased from 74.5 (±5.3) preoperatively to 87.6 (±10.1) postoperatively (*P* = 0.007). For divergent strabismus, the mean Esterman VF score was stable.

### Driving Confidence Questionnaire

Concerning the questionnaire analysis, 90% of patients expressed difficulties with one or more driving maneuvers. The mean self-confidence scores significantly decreased from 20.5 (±10.3) points before surgery to 11.0 (±6.0) points after surgery (*P* < 0.001), with an average variation of 9.5 points between the preoperative and postoperative settings, showing an important improvement of self-confidence during driving. Furthermore, 80% of patients felt that surgery had a beneficial effect on driving.

### Driving Simulator Results (Target Identification and Driving Performance)

Correct identification of road signs is observed before and after surgery. However, the distance at which the road signs are identified is significantly higher after surgery (*P* < 0.001).

The average speed of the vehicle increased significantly after strabismus surgery (*P* = 0.0136). In particular, the speed near the targets (30 m) is higher after surgery (*P* = 0.0123). A significant decrease in ocular movements near targets is also observed (*P* = 0.0072). In addition, the number of brake pedal depressions and the rate of brake pedal depressions slightly decreased after strabismus surgery.

### Learning Effect Analyses

**Figure 1.**
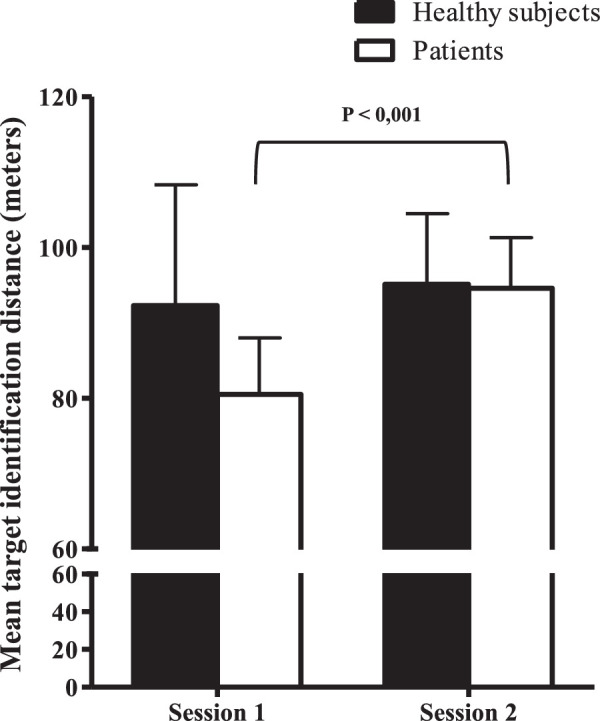
Mean target identification distance for both groups (i.e. healthy subjects and patients) and for both driving sessions. Bars represent standard errors. The *P* value indicates a significant difference for the mean target identification distance between sessions 1 and 2 in the patient group.

**Figure 2.**
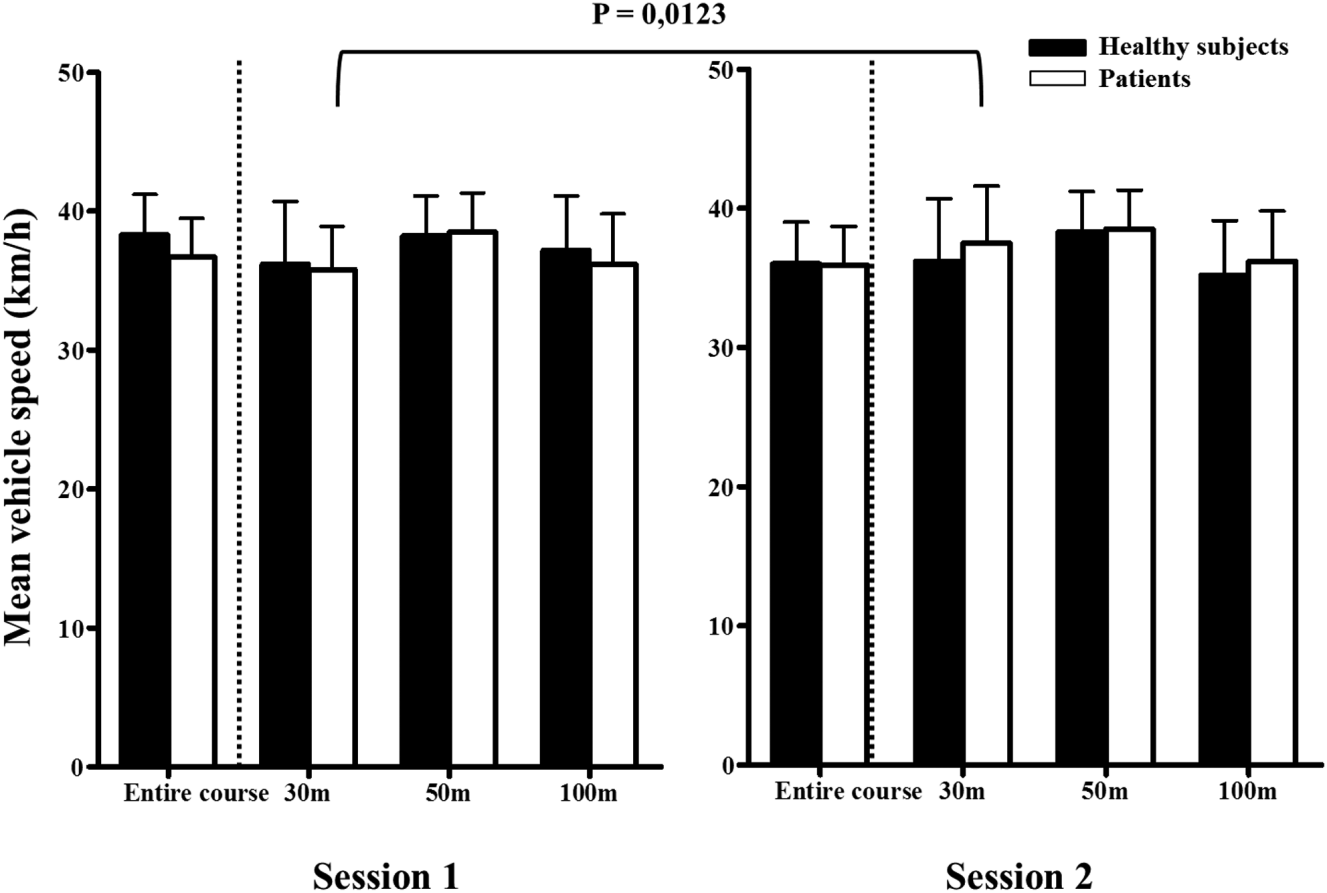
Mean vehicle speed for both groups (i.e. healthy subjects and patients) and for both driving sessions for the three distances to the target (i.e. 30, 50, and 100 meters) and for the entire route. Bars represent standard errors. The *P* value indicates a significant difference for the mean vehicle speed at 30 meters between sessions 1 and 2 in the patient group.

**Figure 3.**
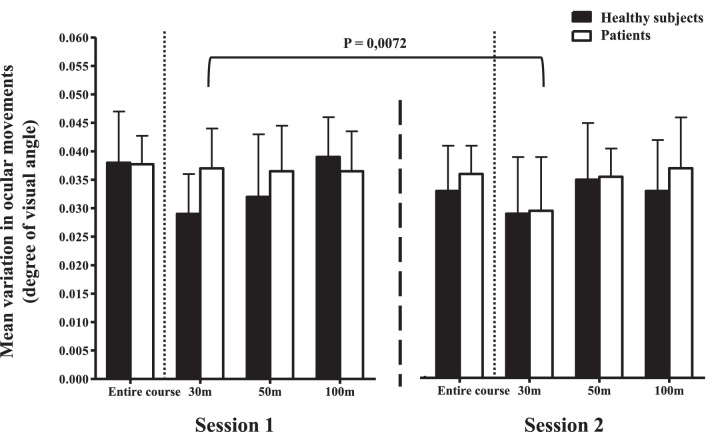
Mean eye movements near targets for both groups (i.e. healthy subjects and patients) during both driving sessions for the three distances to the target (i.e. 30, 50, and 100 meters) and for the entire route. Bars represent standard errors. The *P* value indicates a significant difference for the mean variation in ocular movements at 30 meters between sessions 1 and 2 in the patient group.

**Figure 4.**
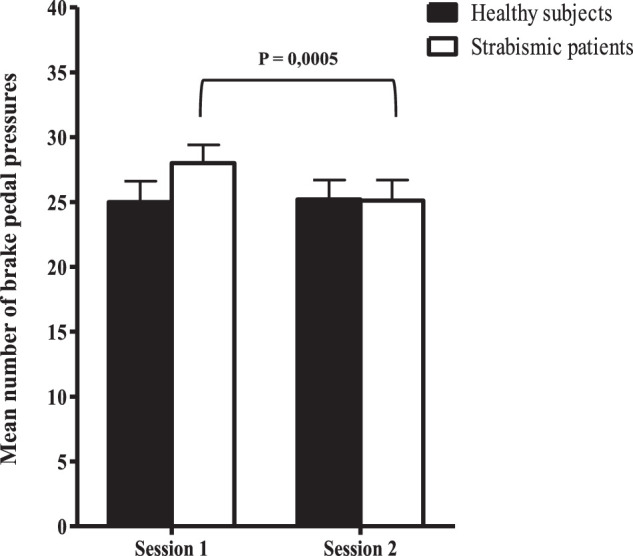
Mean number of brake pedal depression for both groups (i.e. healthy subjects and patients) during both driving sessions. Bars represent standard errors. The *P* value indicates a significant difference for the mean number of brake pedal pressures between sessions 1 and 2 in the patient group.

To distinguish between the changes in driving parameters that would be due to the result of surgical management and those due to a learning effect, we also enrolled non-strabismic controls who performed the same simulator driving test as patients with strabismus. There was no marked change in control group results between the two sessions, showing the absence of learning effect. In addition, patient outcomes improved after surgery and are approaching controls for the following parameters: mean target identification distance (Fig. 1), vehicle speed (Fig. 2), and eye movements near targets (Fig. 3), and brake pedal depressions (Fig. 4).

## Discussion

The present study explored the pre- and postoperative evolution of patients with strabismus and the effect of surgery on driving ability. This study demonstrates the potential beneficial effects of strabismus surgery on driving ability, with significant improvements in self-confidence during driving, visual function, and driving on a simulator. The main weaknesses of the study are related to the use of a simulator compared to driving in a real situation and the limited number of patients, which do not allowed comparison between esotropia and exotropia.

The first study part focused on the feelings of patients with strabismus, clearly revealing a loss of self-confidence when driving. In our study, many patients with strabismus reported experiencing difficulties during driving, or even gave up driving, especially in exceptional situations (night, rain, fog, etc.), which was also the case in previous studies conducted with other ocular diseases.^8^^,^^13^ Strabismus surgery was shown to significantly improve patient self-confidence, as evidenced by a significant improvement in the self-confidence questionnaire for driving. In total, the analysis of self-confidence for the driving questionnaire showed that 80% of patients believed that strabismus surgery had a positive impact on driving ability. This beneficial effect may probably be accounted for a specific improvement in certain visual functions (VF in particular), but also by a nonspecific beneficial effect of surgery.^19^^,^^20^ This enhanced driving confidence is an essential element that should be presented to the patient and taken into account in the surgical decision making.^16^^,^^17^^,^^21^

Regarding functional changes induced by surgery, the binocular visual field was found to increase in case of convergent strabismus, whereas it remained unchanged in case of divergent strabismus. In our study, strabismus surgery did not significantly improve stereoscopic vision, which is probably due to the too low number of patients with maintained binocular potential (low potential for stereoscopic vision because of early childhood strabismus). Moreover, we have not been able to demonstrate the impact of binocular vision on driving, as was the case in previous studies.^3^^,^^15^

Real condition driving proves to be difficult to assess in patients with loss of confidence for methodological and ethical reasons. We have, therefore, decided to assess the patients’ simulator driving capacity while performing a pre-established task before and after surgery by studying real-life parameters (driving speed, line crossing, etc.), but also the target detection capability and eye movement variations while driving in order to obtain a fine and precise representation of patients’ behavior. We believe that this was the first study to use a driving simulator in patients with strabismus. Simulator experiments have previously been performed in other ocular conditions by comparing patients with AMD or glaucoma to healthy subjects. The driving simulator offers the advantage of standardized conditions, presenting a scenario identical to all participants in perfect safety conditions. The simulator tests are also more practical to organize on a single place with identical conditions. In addition, the simulators are particularly suited to the study of eye movements. Conversely, the simulator also has some limitations. The driving conditions are a simplified reproduction of the reality (light conditions, precipitation, number of vehicles on the road …) and does not represent the complexity of the driving in real situation, in particular, because of the graphic aspect presented to the participants. In addition, participants are fully aware of not being in real life, notably in the event of an accident. This can lead to behaviors that are significantly different from those observed under real conditions. Overall, poor simulator performance is not necessarily correlated with driving unfitness. However, the simulator remains an effective tool for measuring various parameters in relation to driving in a reproducible manner. It makes it possible to formulate certain hypotheses on the impact of a visual deficit, in our case, strabismus on vision. Thus, the differences observed on the simulator before and after surgery, associated with the modifications perceived by the patients, suggest a possible improvement of the capacities of driving. If possible, the hypotheses formulated in this work should be supported by a study on the road.

In our study, we chose to compare the driving performance of 20 patients with strabismus before and after surgery. In order to determine the functional deficit induced by strabismus and eliminate any learning effect, we also assessed a group of healthy controls who performed two driving simulator sessions at a 3-month interval. Strabismus surgery has improved the average speed of the course, but has also significantly reduced the number of pressures and the rate of brake pedal depressions. Strabismus surgery also significantly improved the target detection distance in patients. In other words, patients were able to identify a defined target at a greater distance while driving. Late target detection was significantly correlated with increased eye movements and a slowest mean vehicle speed, notably at a 30 meter distance (near to the target), probably because patients with strabismic adapt their viewing behavior by increasing their visual scanning, as previously observed in patients with glaucoma.^12^^,^^22^ This compensation by increased eye movements disappeared after strabismus surgery, which may be related to VF improvements. The improved binocular VF could contribute to this better spatial perception. Moreover, a major contributing factor to vehicle accidents may be the difficulty to efficiently allocate gaze, which may be impaired by attention defects^23^ or strabismus. Strabismus surgery could lead to an improved overall perception while driving, resulting in reduced eye sweeping. Finally, the increase in identification distance may be particularly relevant in terms of road safety, notably in urban areas.

## Conclusions

Overall, this pilot study demonstrates the positive impact of strabismus surgery on driving ability. Significant improvements were observed in terms of driving self-confidence, visual function (enlarged binocular VF), but also simulator-based driving. Studies should be done in a larger cohort and in real-life driving conditions to confirm the benefit of strabismus surgery on driving skills.

## Supplementary Material

Supplement 1
